# Influence of the H1 Antihistamine Mepyramine on the Antibacterial Effect of Florfenicol in Pigs

**DOI:** 10.3390/vetsci8090197

**Published:** 2021-09-16

**Authors:** Gustav G. Bruer, Daria Gödecke, Manfred Kietzmann, Jessica Meißner

**Affiliations:** 1 Chemical Safety and Toxicology, Fraunhofer Institute for Toxicology and Experimental Medicine (ITEM), 30625 Hannover, Germany; 2 Department of Pharmacology, Toxicology and Pharmacy, University of Veterinary Medicine Hannover Foundation, 30559 Hannover, Germany; Daria.Goedecke@tiho-hannover.de (D.G.); Manfred.Kietzmann@tiho-hannover.de (M.K.); Jessica.Meissner@tiho-hannover.de (J.M.)

**Keywords:** *Escherichia coli*, antihistamine, antibacterial, pig

## Abstract

The effect of florfenicol against *Escherichia coli* (*E. coli*) was investigated in vivo to confirm results of an in vitro study of Bruer et al. (2019), which has shown positive effects of various antibacterial agents in combination with the antihistamine mepyramine (MEP). Therefore, pigs were treated in three different settings: An untreated control group, 10 mg/kg florfenicol (FFC) and 10 mg/kg FFC in combination with 20 mg/kg MEP. *E. coli* were isolated from faecal samples and analyzed in growth quantity and resistance to FFC. The FFC medication induced an increased number of resistant *E. coli* strains isolated from faecal samples. The number of colonies detected after cultivation of animal samples treated with 10 mg/kg FFC was higher than the number of colonies after treatment with 10 mg/kg FFC in combination with of FFC and MEP. Furthermore, the effect of both compounds was examined on bacterial susceptibility of *Pasteurella multocida* in vitro, where the combination of FFC with MEP resulted in a diminished minimum inhibitory concentration. We confirmed the development of bacterial resistance in the intestine as non-target tissue caused by the use of the antibacterial agent florfenicol. Moreover, the combination of FFC with an antihistamine like MEP offers a possibility to enhance the efficacy of an antibacterial treatment and modifies the effect on gut microbiota.

## 1. Introduction

Antibiotics are absolutely essential for the treatment of bacterial infections in humans and animals. However, the existing and progressive development of resistance in many microorganisms is alarming [1]. To ensure sufficient efficacy, antibacterials must reach concentrations above the minimum inhibitory concentration (MIC) in the target tissues. In a previous in vitro study, enhanced effects for combinations of various antibacterials with histamine H1 receptor antagonists were demonstrated [2]. H1 receptor antagonists like mepyramine (MEP) represent first-generation antihistamines, commonly used for the treatment of allergic diseases [3]. The in vitro study confirmed results of Pan et al. [4], who could demonstrate in clinical studies with pigs that an antibacterial therapy was positively influenced by additional treatment with antihistamines. The authors concluded that the antihistamines should have positive effects on pathophysiological mechanisms independently of the antibacterial effect.

Florfenicol (FFC) is a synthetic analogue of chloramphenicol, belonging to the group of phenicols. The use of chloramphenicol was banned for treatment of food-producing animals in 1994, but FFC is currently approved for the treatment of respiratory diseases in cattle and pigs, primarily caused by bacteria like *Pasteurella multocida* (*P. multocida*) and *Mannheimia haemolytica* [5]. Phenicols reversibly bind to the 50S subunit of ribosomes, which interferes with bacterial protein biosynthesis and leads to a bacteriostatic effect. They are effective against a broad spectrum of bacteria including *E. coli*. [5,6].

The resistance level not only of pathogens, but also of the commensal microbiota, is increased with each antibacterial treatment [7,8,9]. Considering the one-health-concept, the development of bacterial resistance in non-target tissues (i.e., intestine) caused by the use of antibacterials deserves particular attention. To ensure that bacterial infections can be treated in the future, efforts are being made to reduce the incidence of infections, optimize the use of antibiotics and develop new antimicrobially active agents [10]. The first two activities are considered long term, with attempts to implement them in practice through various approaches [11]. The development of new antibiotics is given a high priority in research; however, only a few new substances have come into the market in recent years [12].

Thus, the aim of the present study was to examine the effect of an antibacterial treatment of pigs with the antibiotic agent FFC in combination with the antihistaminic drug MEP. The antibiotic FFC was chosen because it offered the highest reduction of the MIC for the combination with MEP in vitro [2]. Moreover, the checkerboard method was used to study the effect of the combination treatment (FFC and MEP) on the MIC of a pathogenic porcine *P. multocida* strain. This reallocation of already known active compounds that have a potential effect on bacteria could be another strategy.

## 2. Materials and Methods

### 2.1. Animals

Twelve healthy weaned female and male pigs (hybrid breeding program: boar strain db77^®^, sow strain db. Viktoria) were obtained from the Farm for Education and Research of the University of Veterinary Medicine Hannover. Before housing, the stable was cleaned and disinfected. After dry cleaning, wet cleaning with a high-pressure cleaner followed. Subsequently, a disinfection with Venno^®^ FF super (Menno Chemie Vertrieb GmbH, Norderstedt, Germany) was performed until there was no growth of any bacterial strain on cultivated agar plates according to the method of Römer et al. [13].

At the start of the study, the pigs were 10–12 weeks old. In order to ensure that only pigs without FFC resistant *E. coli* (MIC > 8 µg/mL) in their intestinal microbiota were included in the study, rectal swabs were taken and processed in a microbiology lab. The swabs were separately placed in 5 mL of lysogeny broth (Sifin Diagnostics GmbH, Berlin, Germany) [14] and incubated for 24 h at 37 °C. Subsequently, 100 µL of each incubated sample were spread on endo agar plates (Sifin Diagnostics GmbH, Berlin, Germany) [15] which were supplemented with different FFC concentrations (4, 8, 16 µg/mL).

The pigs were stabled with a day–night rhythm of twelve hours each in addition to daylight. They were randomly divided into four groups and kept in separate pens (1.5 × 3 metres) in the same room without any physical contact between the groups. The sample size was determined based on the experience of previous studies [13,16]. A positive pressure ventilation system was used and the temperature was kept between 23 and 24 °C. The relative humidity ranged from 50 to 60 %. The pigs were kept on straw and fed once a day in the morning with standard porcine feed in a granulated form (Deuka primo pro, Cremer, Düsseldorf, Germany) via feeding trough. Water was available *ad libitum* via nipple drinkers. The pigs were acclimatised for two weeks before the experiment started. During the entire experiment, all animals stayed clinically healthy.

The stable was only entered by humans equipped with disposable protective clothing (overall, gloves and footwear) in order to prevent bacteria transmission from the environment into the stable. All materials required for animal care were made separately available for each group.

After completion of the study, all animals were euthanized with pentobarbital (i.v., 80 mg/kg bw). The study was authorised by the Lower Saxony State Office for Consumer Protection and Food Safety, Lower Saxony, Germany (reference number 33.19-42502-04-16/2067).

### 2.2. Study Protocol

The groups of animals (*n* = 4) were treated as follows: 

Group 1: untreated control, received unmedicated feed.

Group 2: Treatment with FFC, 10 mg/kg bw via feed over five days.

Group 3: Treatment with FFC, 10 mg/kg bw FFC combined with MEP, 20 mg/kg bw via feed over five days.

FFC was applied as Floron^®^ 40 mg/g (TAD Pharma, Cuxhaven, Germany) at the recommended dosage of 10 mg/kg bw. Mepyramine maleate (pure substance, Sigma-Aldrich, St. Louis, MO, USA) was used for MEP medication. Since there is no specific recommendation for the dosage of mepyramine in pigs, we carried out preliminary studies with the result that the pigs tolerate the drug well at a dosage of 20 mg/kg.

All groups were fed with the same standard pig feed supplemented with the different medications (see above) over five days in the morning via feeding trough (animal/feeding-place ratio 1:1). The total feed intake of the animals was controlled. Rectal faecal samples were taken the day before the first administration of medicated feed (day 0), as well as one day after the last day of treatment (day 6). The study was conducted in an unblinded manner, among others to avoid any mix-up.

### 2.3. Isolation and Screening of E. coli from Faeces

One gram of each faecal sample was serially diluted to 10^−7^ in 9 mL tryptone sodium chloride solution (0.1 % tryptone, 0.85 % NaCl). Next, 100 µL of all dilutions were spread out on endo agar plates without FFC supplementation and on endo agar plates supplemented with 4, 8 and 16 µg/mL FFC [17]. Plates were incubated at 37 °C for 20–24 h. Ten lactose-fermenting coliform colonies per animal and plate were picked using a self-produced template, which localised ten fix points on every plate to avoid subjective selections. Isolates were verified as *E. coli* using LMX broth according to Manafi and Ossmer [18] and indole reaction using Kovacs reagent [15]. Samples with uncertain results were confirmed as *E. coli* by RapID™ ONE System (Thermo Fisher Scientific, Waltham, MA, USA) according to the manufacturer’s instructions. The determined MIC values measured by FFC supplemented agar plates were verified with FFC MIC Test Strips (Liofilchem^®^, Waltham, MA, USA). Colonies on agar plates supplemented with 16 µg/mL FFC showed a resistance above the limit of determination (MIC > 256 µg/mL).

### 2.4. Checkerboard Experiments

As described already in a previous publication [2], the in vitro checkerboard method was used to show if MEP is able to increase the FFC efficacy against bacteria. A *P. multocida* strain (*P. multocida* 1117/1/19, kindly provided by Dr. Jutta Verspohl, Institute for Microbiology, University of Veterinary Medicine Hannover) was used for checkerboard experiments. The *P. multocida* strain was isolated from lung tissue of pigs suffering from bronchopneumonia and was susceptible for FFC (MIC ≤ 1 µg/mL) [17].

The bacterium was subcultured on 7% Columbia sheep blood agar plates (Thermo Fisher Scientific, Waltham, MA, USA) and incubated for 24 h at 37 °C. The *P. multocida* strain was diluted in M9 media. FFC (Cayman Chemical Company, Ann Arbor, MI, USA) was dissolved in 10 µL dimethyl sulfoxide. The checkerboard tests were carried out, according to Bruer et al. [2]. The experiment was performed six times. Dose reduction indices (DRI) were calculated by means of: DRI = MIC_alone_ /MIC_combined_ [19]. The aim was to demonstrate an increased/positive effect of MEP in combination with FFC against a pathogenic bacterium.

### 2.5. Statistical Analysis

Statistical evaluations were calculated with GraphPad Prism software (version 8.0.1, GraphPad Software, Inc., La Jolla, CA, USA). Medians of MIC values from *E. coli* and *P. multocida* isolates for treatment alone and in combination were compared using the Mann–Whitney U-test. Colony counts of the control group compared with the treated groups were assessed using the Mann–Whitney U-test, as well. Statistical significance was set at a *p* value < 0.05.

## 3. Results

### 3.1. In Vivo Study

Rectal faecal samples were taken to evaluate the treatment effect of FFC and FFC combined with MEP on the commensal microbiota of pigs. On day 0, coliform colonies were found on all plates of the 10^−3^ dilution, as well as on day 6 in the controls (group 1). There was no coliform growth on the plates supplemented with 4, 8 and 16 µg/mL FFC.

**Figure 1:**
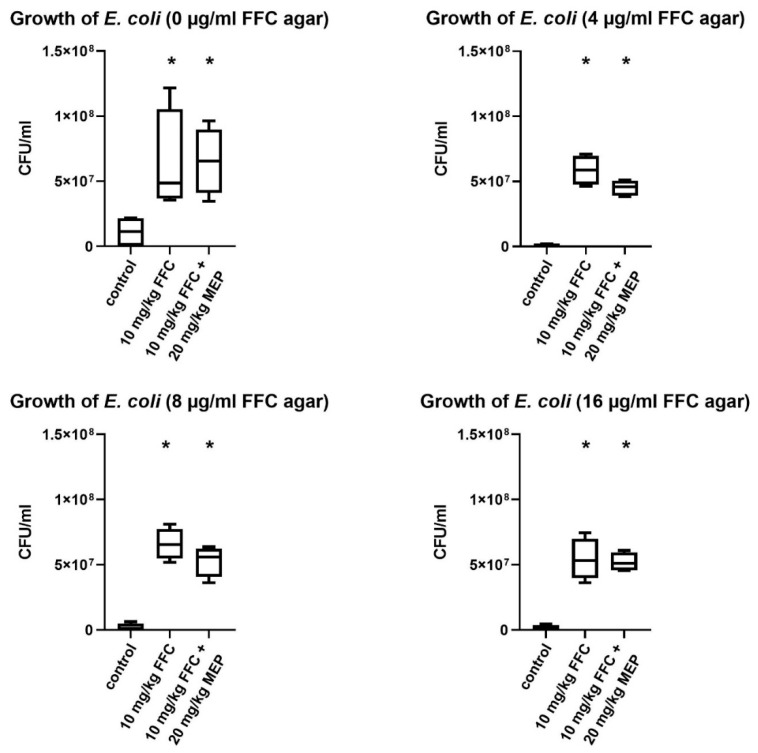
CFU/mL of *E. coli* for each group of pigs cultured on supplemented agar plates on day 6 after five days of medication. Data are expressed as Tukey box plots. * = *p* < 0.05 (when compared against control group 1 using Mann–Whitney U-test).

To compare the treatment groups on day 6 after the five-day-medication period, the 10^−5^ and 10^−6^ dilutions were used to calculate CFU/mL (colony forming unit). For the medicated groups, a change in bacterial growth appeared on day 6. Compared to the control group, enhanced bacterial growth was found on agar plates without supplementation of FFC on day 6 in samples of all treatment groups (group 2 and 3). To find out if these bacteria were FFC susceptible, all bacteria were also cultivated on agar plates supplemented with 4, 8, 16 µg/mL FFC. The results of the growth of *E. coli* on the plates supplemented with 16 µg/mL FFC demonstrated that the cultivated bacteria were resistant against FFC since the European Committee on Antimicrobial Susceptibility Testing 

(EUCAST) defined the epidemiological cutoff (ECOFF) of *E. coli* with 16 µg/mL FFC [17]. In samples of pigs treated with 10 mg/kg FFC (group 2), about 5x10^7^ CFU/mL (median) were counted, while in the samples of animals treated with 10 mg/kg FFC in combination with 20 mg/kg MEP, the number of CFU/mL was lower (median) for the 4 µg/mL and 8 µg/mL FFC agar screening (Figure 1).

For the treated groups, the consistency of the faeces varied between pasty and mostly watery. This effect was observed partly in our experiments. Otherwise, all pigs were clinically healthy during the entire experiment.

### 3.2. Checkerboard Experiments

Table 1 shows the MIC values of the *P. multocida* isolate. In comparison with single treatment with FFC, the MIC value of FFC against the *P. multocida* isolate 1117/1/19 was diminished by the combination with MEP. A DRI of 2 (range 2–4) was calculated.

## 4. Discussion

A study by De Smet et al. [16] demonstrated that the susceptible *E. coli* population in the pig’s intestine shifted to a resistant one as a consequence of FFC administration. This resistance development of the intestinal microbiota during FFC treatment was confirmed in the present study. The number of commensal *E. coli* in the intestine of pigs extremely increased under FFC medication over five days. Only resistant *E. coli* colonies can grow on agar plates with the addition of 16 µg/mL FFC. It can be proposed that, due to selection of FFC treatment, the microbiota shifted to a FFC resistant one.

It is unclear whether the increase in cultivated resistant *E. coli* is due to mutation. Likewise, it could be possible that resistant colonies were already present in the intestines of the pigs, which were able to multiply strongly due to the selection pressure of FFC. However, resistant colonies were not detected by the preselection of the animals before starting the experiment.

Numerous studies have shown that the use of antibacterials increases the resistance of pathogenic bacteria, as well as the commensal microbiota [7,8,9]. The results of the present study and those of De Smet et al. [16] suggest similar findings related to FFC. The question arises as to whether resistant *E. coli* in the intestine of the pigs treated with FFC influences the animal health or its environment. Saenz et al. [20] conducted a study about the effects of FFC on the gut microbiota in fish. Their results suggest that an oral administration of antibacterials increases the potential for the exchange of antibiotic resistance genes in the gut. They also showed an enrichment of these genes in the fish environment. Moreover, shifts in the gut microbiota towards well-known putative pathogens following FFC treatment were observed by Saenz et al. [20]. Thus, resistant commensal bacteria represent a potential resistance reservoir in the intestine as well as the environment [7,8,9], which poses a high risk for resistance transmission.

Drug levels above a mutant prevention concentration (MPC) are necessary to restrict the mutation causing the development of bacterial resistance [13]. The MPC is the lowest concentration preventing growth at a high inoculum using agar dilution methodology [21]. 

It represents a difficult task for scientific studies that the intestinal microbiota varies inter-individually due to the influence of different parameters like nutrition, genetics and habitat [22,23,24,25], as well as shown in previous studies for *E. coli* [13,26].

In the present study, no genetic analysis of the bacteria was carried out that could show potential resistance development (e.g., virulence factors). Thus, further studies are required.

The results of this study demonstrate a tendency that the number of colonies found after cultivation on agar with addition of 4 and 8 µg/mL FFC was higher using samples from animals treated with 10 mg/kg FFC compared with the combination treatment of FFC/MEP. The number of resistant bacteria selected by the combination treatment is diminished. This confirms in vitro findings of Bruer et al. [2] on the dose reduction of antibacterials in combination with MEP.

Potential mechanisms for the effect of antihistamines in combination with antibiotics have already been discussed by El-Banna et al. [27] and Bruer et al. [2]. The mechanism could be based on the inhibition of bacterial efflux pumps by the antihistamine. Inhibition of biofilm formation would also be possible [27]. The antihistamine could also change the membrane permeability of the bacteria due to its surfactant-like properties [28,29,30]. Furthermore, an interaction with the base pairs of the bacterial DNA [31] or an elimination of the protective mechanism of the bacteria against stress through an acidic environment is being discussed [2].

One indication of FFC in pigs is the treatment of respiratory diseases caused by *P. multocida*. In order to check a possible enhanced efficacy in vitro against *P. multocida*, a checkerboard study was performed in accordance to a previous study with commensal *E. coli* [2]. The combination of FFC and MEP showed a significant reduction of the MIC against *P. multocida*. By using combinations of FFC and MEP in the checkerboard experiment, the MIC values were significantly reduced and ranged between 0.125 and 0.5. The calculated DRI was 2 (Table 1). Thus, a next step could be proving the clinical relevance of this result in further in vivo studies, since only healthy pigs were included in the present study to examine the effect on commensal intestinal bacteria.

## 5. Conclusions

In case of a FFC therapy, the possible resistance development in the commensal microbiota in pigs has to be taken into account, thus demonstrating once again the importance of a prudent use of antibacterials. For this purpose, the combined use of FFC with an antihistaminic agent like MEP could be a possible alternative to reduce the development of resistance of the bacteria in the targeted or non-targeted tissue. Due to the results of the present study, a resistance development in commensal intestinal bacteria cannot be prevented by a combination of FFC with MEP, although the amount of resistant bacteria was diminished. Consequently, further studies are required to study a beneficial effect of a combination treatment on pathogenic bacteria like *P. multocida*.

## Figures and Tables

**Table 1:** MIC of FFC in combination with MEP for *P. multocida*.

FFC in Combination with MEP	MIC (µg/mL)				DRI Median	DRI Range
	Alone		Combined			
	Median	Range	Median	Range		
*P. multocida* 1117/1/19	0.5	0.5–1	0.25 *	0.125–0.5	2	2–4

Data are expressed as median and range (*n* = 6), FFC: florfenicol, MEP: mepyramine. MIC: minimum inhibitory concentration. DRI: dose reduction index (MIC alone/MIC combined). *: Significantly different (*p* < 0.05).

## Data Availability

The datasets used and/or analyzed during the current study are available from the corresponding author on reasonable request.
